# Retinal Vascular Occlusion Following COVID-19 Vaccination: A Comprehensive Review of Observational Study and Pathophysiological Mechanisms

**DOI:** 10.3390/vaccines13070733

**Published:** 2025-07-07

**Authors:** Yuchen Zhang, Haoliang Zhang, Kangjia Lv, Xin Lin, Feng’e Chen, Hui Cao, Chong Chen

**Affiliations:** 1 Department of Ophthalmology, Shanghai General Hospital, Shanghai Jiao Tong University School of Medicine, Shanghai 200080, China; zhang.yu.chen@sjtu.edu.cn (Y.Z.); dasein@sjtu.edu.cn (H.Z.); lvkangjiasjtu@sjtu.edu.cn (K.L.); xin_lin@fjmu.edu.cn (X.L.); fenge.chen@shgh.cn (F.C.); 2 National Clinical Research Center for Ophthalmic Diseases, Shanghai 200080, China; 3 Shengli Clinical Medical College of Fujian Medical University, Fujian Medical University, Fuzhou 350000, China; 4 Department of Ophthalmology, Fujian Provincial Hospital, Fuzhou University Affiliated Provincial Hospital, Fuzhou 350000, China; 5 Harvard Retinal Imaging Lab, Massachusetts Eye and Ear, Harvard Medical School, Boston, MA 02115, USA

**Keywords:** COVID-19 vaccination, retinal vascular occlusion (RVO), retinal artery occlusion (RAO), vaccine-induced immune thrombotic thrombocytopenia (VITT), pathophysiological mechanisms, mRNA vaccines, adenoviral vector vaccines, spike protein, PF4 antibodies

## Abstract

**Background**: Retinal vascular occlusion (RVO) and retinal artery occlusion (RAO) have been reported as rare adverse events following COVID-19 vaccination, raising concerns about vaccine safety. This review synthesizes cohort and case–control studies assessing the association between COVID-19 vaccines and RVO/RAO, while exploring potential pathophysiological mechanisms. **Methods**: We analyzed large-scale population-based studies from South Korea, Europe, and the TriNetX database, focusing on odds ratios (OR), hazard ratios (HR), and relative risks (RR) across mRNA and adenoviral vector vaccines. Pathological processes were hypothesized based on molecular and clinical evidence. **Results**: Studies investigating the association between COVID-19 vaccination and retinal vascular occlusion show conflicting results; some studies report no association (e.g., OR 0.93, 95% CI 0.60–1.45), others suggest reduced risk (e.g., OR 0.80, 95% CI 0.64–0.99), and one indicates increased risk over two years (HR 2.19, 95% CI 2.00–2.39). Adenoviral vector vaccines, particularly ChAdOx1, show higher RAO incidence in specific cohorts. Proposed mechanisms include vaccine-induced immune thrombotic thrombocytopenia (VITT) via anti-PF4 antibodies, spike protein-mediated endothelial dysfunction, and adjuvant-driven inflammation. **Conclusions**: While causality remains unproven, temporal heterogeneity and vaccine type-specific risks warrant further investigation. Longitudinal studies with robust controls are needed to clarify these associations in the post-pandemic context.

## 1. Research Background

### 1.1. Prevalence and Hazards of COVID-19

Initiated in March 2020, the World Health Organization (WHO) classified COVID-19 as a global public health emergency of international concern (PHEIC), resulting in considerable morbidity and mortality. SARS-CoV-2 is a β-subclass, single-stranded, positive-stranded RNA virus with an envelope and round or oval particles [1] that employs its S protein as a critical structure for binding to host cells via the angiotensin-converting enzyme 2 (ACE2) receptor, leading to a spectrum of respiratory symptoms and disease severity [2]. Ocular manifestations of COVID-19 primarily include inflammation of the conjunctiva, cornea, and sclera, along with uveitis and retinal and optic nerve damage. Retinal vascular occlusion has garnered significant attention from the research community, yet no definitive conclusions have been reached [3,4,5]. A literature review [6] of cases reporting severe acute respiratory syndrome-coronavirus-2 (SARS-CoV-2) pneumonia reported before January 2022 suggested it as a rare but possible manifestation of the disease. A retrospective cohort study [7] of 1,460,634 individuals found that patients with new-onset COVID-19 had a significantly higher risk of branch retinal vein occlusion (BRVO) compared to uninfected controls (hazard ratio [HR] 1.27, 95% confidence interval [CI] 1.04–1.52). Contrasting epidemiological patterns emerged from South Korean surveillance [8]; a modest decline in retinal vein occlusion rates contrasted with rising retinal artery occlusion frequency during COVID-19 surges, though confirmed SARS-CoV-2 infection exhibited no causal association with either condition.

### 1.2. Vaccine Development, Rationale, and Vaccination Status

The genetic sequence of SARS-CoV-2 was first published on 11 January 2020 [9]. Vaccines against SARS-CoV-2 are the most effective means for controlling the COVID-19 pandemic, developed in a remarkably short timeframe. These include mRNA vaccines, which deliver the genetic code via lipid nanoparticles (e.g., Pfizer/BioNTech (Pfizer Inc. & BioNTech SE, New York, NY, USA, Modern (Moderna, Inc., Cambridge, MA, USA))); adenoviral vector vaccines, which use genetically modified adenoviruses to insert the spiky protein genes (e.g., Janssen (Janssen Vaccines, Leiden, The Netherlands), Oxford—AstraZeneca (AstraZeneca PLC, Cambridge, UK))); and whole virus vaccines, produced by inactivating the virus chemically or physically (e.g., Sinopharm (China National Pharmaceutical Group (Sinopharm), Beijing, China, CoronoVac (Sinovac Biotech Ltd., Beijing, China))). Extensive multicenter clinical trials confirmed their safety prior to market authorization. As of December 2022, over 13 billion doses of the COVID-19 vaccine had been administered globally. However, reports of thrombocytopenia and thromboembolic events, particularly with adenoviral vector vaccines, have raised safety concerns [10,11,12]. Despite the low incidence of these adverse events, European countries suspended the use of the Oxford–AstraZeneca vaccine due to its association with high mortality rate [13]. This has heightened public apprehension. Healthcare professionals have since intensified efforts to closely monitor and report the post-vaccination adverse effects.

### 1.3. Retinal Vein Occlusion and Retinal Artery Occlusion

Retinal vein occlusion (RVO), the second most prevalent retinal vascular disease, is a leading cause of vision impairment. In 2015, RVO affected over 28.06 million people worldwide, with a prevalence rate of 0.77% among those aged 30–89 [14]. In China, the prevalence of retinal vein occlusion (RVO) significantly increased from 0.099% (95% CI, 0.073–0.126) in 2019 to 0.188% (95% CI, 0.142–0.244) in 2021 [15]. Fundus findings in RVO patients include disk swelling, retinal hemorrhages, tortuous dilated veins, and cotton wool spots. Vison loss may result from complications such as macular edema and macular ischemia, which are persistent and challenging to treat [16,17]. RVO is often associated with systemic inflammation or localized inflammatory ocular disorders, including but not limited to systemic vasculitis, Behçet’s disease, systemic lupus erythematosus, Takayasu’s aortitis, polyarteritis nodosa, and antiphospholipid syndrome. Among localized inflammatory ocular conditions, idiopathic retinal phlebitis is the most common. Non-inflammatory predisposing factors encompass systemic diseases (e.g., hyperhomocysteinemia, hypertension, diabetes mellitus, dyslipidemia, smoking, renal disease) and localized ocular diseases (e.g., glaucoma, hypertensive retinopathy) [18]. Primary RVO, lacking an identifiable etiology beyond these factors, is attributed to the Virchow triad (vascular wall damage, blood stasis, and hypercoagulability) [17,19].

Retinal artery occlusion (RAO) denotes sudden cessation of arterial retinal blood flow due to thromboembolism or vasospasm, causing acute monocular vision loss. This spectrum includes branch (BRAO) and central (CRAO) subtypes. CRAO incidence ranges 1–2/100,000 annually with male predominance, while BRAO constitutes ~33% of RAO cases [20]. Visual deficit severity correlates with occluded vessel caliber and ischemic duration. RAO pathogenesis parallels ischemic stroke, principally involving atherosclerosis, cardiogenic/aortic embolism, vasculitides (e.g., giant-cell arteritis), or hypercoagulable states [16].

## 2. Cohort and Case–Control Studies: Is RVO or RAO Associated with COVID-19 Vaccines?

Few cohort studies provide odds ratio (OR) on the association between COVID-19 vaccines and retinal vascular occlusion (Table 1). Results vary widely; most studies concluded that there was no association, some suggested the vaccine significantly reduced the risk, and others indicated potential risk.

**Figure 1:**
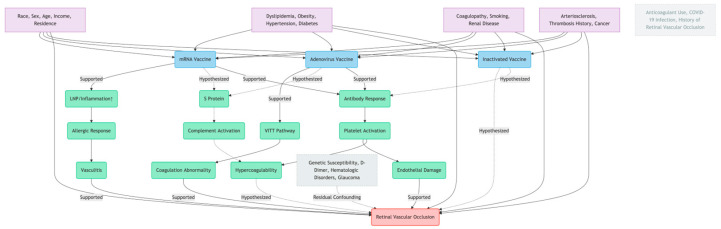
Arrows denote causal directions (exposure→mediator→outcome). Four confounder groups (demographics/pre-existing conditions, indicated by purple boxes) affect both vaccine exposure (indicated by blue boxes) and RVO risk. Unmeasured variables (dashed boxes) indicate residual confounding. Green boxes represent possible pathologicalmechanisms.Excluded factors were intentionally controlled in some of studies. DAG guides bias adjustment and mechanistic analysis.

This causal diagram (Figure 1) illustrates pathways linking COVID-19 vaccines to retinal vascular occlusion.

Three South Korean studies analyzed national databases (NHIS/KDCA) covering millions of participants, assessing 60-day post-exposure outcomes. Vaccines comprised adenoviral (ChAdOx1, Ad26.COV2.S) and mRNA platforms (BNT162b2, mRNA-1273).

The nationwide population-based cohort study of Kim et al. included 2,742,065 individuals aged  ≥  20 years who received SARS-CoV-2 vaccination, matched 1:10 with unvaccinated controls by gender and age. Their findings established that vaccination lowered the risk of retinal vascular occlusion, with an OR of 0.80 (95% CI, 0.64–0.99; *p*  =  0.039). For those under 40, the protection was even stronger (OR 0.35 for age 20–39, 0.83 for age 40–64, 0.81 for age  ≥  65; *p* for interaction  =  0.028) [21]. Hwang et al.’s self-controlled case series analyzed 6590 RAO and 70,120 RVO cases, reporting incidence rate ratios (IRRs) (95% CI) of 0.95 (0.88–1.01) and 0.96 (0.94–0.98) during the early risk period (0–28 days), and 0.96 (0.89–1.03) and 0.93 (0.91–0.96) in the late risk period (29–56 days), respectively. They found no significant association [22]. Park et al. [23] stratified cohorts into unvaccinated/uninfected (G1), COVID-19-positive (G2), and vaccinated (G3). Within 60 days, RVO incidence was lowest in G1 (0.0072%, 195/2,680,438), intermediate in G2 (0.0114%, 66/577,471), and highest in G3 (0.0159%, 822/5,160,681) (G1 vs. G2: *p* = 0.001; G1 vs. G3: *p* < 0.001; G2 vs. G3: *p* = 0.009). Notably, vaccinated males exhibited significantly reduced RVO risk versus G1 (aHR 0.77, 0.61–0.99; *p* = 0.042).

**Table 1:** This table systematically presents the current evidence base through big data analyses conducted to date investigating the potential association between retinal vascular occlusion and COVID-19 vaccine administration.

Paper	Research Method	Population	Interval Between Onset of Illness	Database	Group Design	Confounders Adjusted	Baseline Characteristics	Excluded	Vaccine	Number of Events/Sample Size (Cumulative Incidence %; 95% CI)	Cumulative Incidence of Vaccine Type	Odds Ratio OR Confidence Interval (95% CI)
Li JX et al. [24]	Nationwide population-based retrospective cohort study	4,619,499 745,041 vaccinated and 3,874,458 unvaccinated	12 weeks or 2 years	TriNetX global network	Employed multivariable-adjusted Cox proportional hazards models after performing a 1:1 propensity score matching between the vaccinated and unvaccinated cohorts.	age, sex, race, comorbidities, medications and previous hospitalization	vaccinated group Age: 52.5 ± 18.5 years; unvaccinated group Age: 52.2 ± 18.2 years.	1. Any diagnosis of retinal vascular occlusion six months prior to the index date. 2. Cases with confirmation of COVID-19 diagnosis. 3. Cases with the use of any antiplatelets, anticoagulants, diuretics, contraceptives, or antihemorrhages 4 weeks prior to the index date.	BNT162b2 (Pfizer), mRNA-1273 (Moderna), Ad26.COV2.S (Janssen)	In 2 years Vaccinated: 3213/743,505 (0.432%; 95% CI, 0.417–0.447%) Unvaccinated: 1599/743,505 (0.215%; 95% CI, 0.205–0.226%)	2 years: First dose of BNT162b2: 120/111,491 (0.108%; 95% CI, 0.089–0.127%) Second dose of BNT162b2: 116/96,135 (0.121%; 95% CI, 0.100–0.143%) First dose of mRNA-1273: 114/50,382 (0.226%; 95% CI, 0.186–0.267%) Second dose of BNT162b2: 106/47,536 (0.223%; 95% CI, 0.182–0.265%) 12 weeks: First dose of BNT162b2: 40/111,491 (0.036%; 95% CI, 0.025–0.047%) Second dose of BNT162b2: 40/96,135 (0.042%; 95% CI, 0.030–0.055%) First dose of mRNA-1273: 32/50,382 (0.064%; 95% CI, 0.043–0.086%) Second dose of BNT162b2: 33/47,536 (0.069%; 95% CI, 0.047–0.093%)	HR 2 years 2.19 (95% CI, 2.00–2.39) HR 12 weeks 3.54 (95% CI, 3.03–4.11)
Dorney, I. et al. [25]	Nationwide population-based retrospective cohort study	3,108,829 patients receiving the mRNA COVID-19 vaccine 718,400 Tdap vaccine 1,470,351 Influenza vaccine	21 days	TriNetX global network	Group 1 received the first dose of mRNA COVID-19 from 15 December 2020 to 15 June 2022; group 2 received the second dose of mRNA COVID-19 vaccine from 15 December 2020 to 15 June 2022; group 3 received influenza vaccine from 1 June 2018 to 31 December 2019; and group 4 received Tdap vaccine from 1 June 2018 to 31 December 2019.	demographic characteristics (age, sex, race and ethnicity) and comorbidities (diabetes, hypertension, and hyperlipidemia)	Age at vaccination: 50.7 ± 20.4 years; Sex: 56.4% female	Any history of encounter diagnoses of RVO any time before the vaccination event	mRNA COVID-19 vaccination	Vaccinated: 104/3,108,829 (0.003%; 95% CI, 0.003–0.004%)	21 days First dose of mRNA vaccine: 45/1,180,006 (0.004%; 95% CI, 0.003–0.005%) Second dose of BNT162b2: 93/1,180,006 (0.006%; 95% CI, 0.008–0.010%)	First dose of COVID-19 mRNA vaccination vs. influenza vaccination (RR, 0.74; 95% CI, 0.54–1.01) First dose of COVID-19 vaccination vs. Tdap vaccination (RR, 0.78; 95% CI, 0.44–1.38), First dose of COVID-19 vaccination vs. second dose of COVID-19 vaccination (RR, 2.25; 95% CI, 1.33–3.81)
Kim, Y. et al. [21]	Nationwide population-based retrospective cohort study	2,742,065 vaccinated 262,603 unvaccinated	60 days	The National Health Insurance Service (NHIS)–COVID-19 cohort database	Vaccinated individuals against SARS-CoV-2. unvaccinated individuals matched at a ratio of approximately 1:10 by gender and age.	sex, age, income, residence, disability, and CCI scores.	age: 48.7 ± 17.1 years; sex: 395,211 (50.9%) male	1. A history of retinal vascular occlusion 2. Aged < 20 years	ChAdOx1 (Astr-Zeneca), BNT162b2 (Pfizer), mRNA-1273 (Moderna), Ad26.COV2.S (Janssen)	Vaccinated: 93/262,603 (0.035%; 95% CI, 0.029–0.043%) Unvaccinated: 725/2,479,462 (0.029%; 95% CI, 0.027–0.031%)	ChAdOx1 503/710,004 (0.071%; 95% CI, 0.065–0.077%) BNT162b2 162/1,292,421 (0.013%; 95% CI, 0.011–0.015%) mRNA-1273 44/388,831 (0.011%; 95% CI, 0.008–0.015%) Ad26.COV2.S 16/88,206 (0.018%; 95% CI, 0.011–0.029)	1. Vaccination lowered the risk of retinal vascular occlusion. OR = 0.80 (95% CI, 0.64–0.99; *p* = 0.039) 2. For individuals aged < 40 years, the vaccination lowered the risk of retinal vascular occlusion occurrence significantly compared with those over the age of 40. Age 20–39, OR = 0.35; Age 40–64 OR = 0.83; Age ≥ 65 OR = 0.81; *p* for interaction = 0.028)
Park, H.S. et al. [23]	Nationwide population-based cohort study.	8,418,590	60 days	The Korea Disease Control and Prevention Agency (KDCA) and the Korean National Health Insurance Service (NHIS)	Group 1 included patients who neither received a diagnosis of COVID-19 nor were vaccinated against COVID-19 (n = 2,680,438) before 1 September 2021. Group 2 included patients who received a diagnosis of COVID-19 (n = 577 471) once or more before 31 January 2022. Group 3 included patients vaccinated at least once (n = 5,160,681) before 1 September 2021.	age, sex, and the presence of hypertension, DM, or dyslipidemia	group 1 age: 33.5 ± 13.3 years; group 2 age: 41.7 ± 21.6 years; group 3 age: 54.2 ± 17.1 years. The population of group 1 was younger, was predominantly male, and had fewer comorbidities, including hypertension and DM, compared with that of groups 2 and 3.	1. A diagnosis of RAO or RVO before the preset index date of each group. 2. Patients with incomplete data regarding the first vaccination (n = 32 269) or demographic data (such as sex and age; n = 943)	ChAdOx1 (AstraZeneca), BNT162b2 (Pfizer), mRNA-1273 (Moderna), Ad26.COV2 S (Janssen)	60 days RAO group 1: 13/2,680,438 (0.0005%; 95% CI, 0.0003–0.0008%) group 2: 4/577,471 (0.0007%; 95% CI, 0.0002–0.0018%) group 3: 73/5,160,681 (0.0014%; 95% CI, 0.0011–0.0018%) 60 days RVO group 1: 195/2,680,438 (0.0072%; 95% CI, 0.0063–0.0083%) group 2: 66/577,471 (0.01143%; 95% CI, 0.0089–0.0144%) group 3: 822/5,160,681 (0.0159%; 95% CI, 0.0149–0.0170%)	In women who received mRNA-1273 vaccines, who showed a higher RAO HR (4.65; 95% CI, 1.27–17.03; *p* = 0.021).	Using incidence rates of group 1 as a reference RAO: Vaccination male group, HR 0.82 (95% CI, 0.53–1.17, *p*-value = 0.644) Vaccination female group, HR 1.37 (95% CI, 0.42–4.44, *p*-value = 0.602) RVO: Vaccination male group, HR 0.77 (95% CI, 0.61–0.99, *p*-value = 0.042) Vaccination female group, HR 1.03 (95% CI, 0.79–1.35, *p*-value = 0.807)
Hwang, S. et al. [22]	Nationwide population-based self-controlled case series	6590 cases of incident RAO 70,120 cases of incident RVO	early risk period (0 to 28 days from vaccination) late risk period (29 to 56 days from vaccination)	Korea National Health Insurance Service (NHIS) and Korea Disease Control and Prevention Agency (KDCA)	Any period immediately following vaccination was designated as a risk period. Any other time during the study period was considered as an unexposed control period. Pre-risk period (−28 to −1 days from vaccination) Risk period, early (0 to 28 days from vaccination) Risk period, late (29 to 56 days from vaccination)	/	RAO: Age 67.69 ± 13.28; Sex: 4025 (61.08%) male. Income level: Low 1730 (26.25%); Previous disease history: Cerebro-cardiovascular 2237 (33.95%), Rheumatic autoimmune 1056 (16.02%). RVO: Age: 63.76 ± 13.88; Sex: 32,318 (46.09%) male; Income level Low 19,395 (27.66%); Previous disease history Cerebro-cardiovascular 16,288 (23.23%), Rheumatic autoimmune 9759 (13.92%).	1. A history of the disease within the 6 years before the observation period 2. Passed away during the study period 3. Missing vaccination or demographic data.	ChAdOx1 (AstraZeneca), BNT162b2 (Pfizer), mRNA-1273 (Moderna), Ad26.COV2 S (Janssen)	RAO: Control period: 3885/1,711,590 (0.227%; 95% CI, 0.220–0.234%) Pre-risk period: 819/380,402 (0.215%; 95% CI, 0.201–0.230%) Risk period, early: 1040/497,916 (0.209%; 95% CI, 0.196–0.222%) Risk period, late: 846/408,542 (0.207%; 95% CI, 0.193–0.221%) RVO: Control period: 40,916/17,900,627 (0.229%; 95% CI, 0.227–0.231%) Pre-risk period: 8399/4,110,437 (0.204%; 95% CI, 0.200–0.208%) Risk period, early: 11,572/5,422,603 (0.213%; 95% CI, 0.209–0.217%) Risk period, late: 9233/4, 470,933 (0.207%; 95% CI, 0.203–0.211%)	/	0–28 days postvaccination, RAO IRR 0.95 (0.88–1.01), RVO IRR0.96 (0.94–0.98) 29–56 days RAO IRR 0.96 (0.89–1.03), RVO IRR 0.93 (0.91–0.96)
Pellegrini, M. et al. [26]	multicenter self-controlled case series	210 RVOs patients	28 days	Five tertiary referral centers in Italy	Days 1–14 after first dose days 14–28 after first dose days 1–28 after first dose days 1–14 after second dose days 14–28 after second dose days 1–28 after second dose all other observed time	/	Age: 68.8 ± 12.3 years; Sex: 111 (52.9%) males	1. Diagnosis of RVO prior to the study period 2. Unavailability of data regarding COVID-19 vaccination status.	ChAdOx1 (AstraZeneca), BNT162b2 (Pfizer), mRNA-1273 (Moderna), Ad26.COV2 S (Janssen)	/	/	Days 1–14 after first dose, IRR 0.87 (0.41–1.85) days 14–28 after first dose, IRR 1.01 (0.50–2.04) days 1–28 after first dose, IRR 0.94 (0.55–1.58) days 1–14 after second dose, IRR 1.21 (0.62–2.37) days 14–28 after second dose, IRR 1.08 (0.53–2.20) days 1–28 after second dose, IRR 1.16 (0.70–1.90)
Paper	Research Method	Population	Interval Between Onset of Illness	Database	Group Design	Confounders Adjusted	Baseline Characteristics	Excluded	Vaccine	Number of Events/Sample Size (Cumulative Incidence %; 95% CI)	Cumulative Incidence of Vaccine Type	Odds Ratio OR Confidence Interval (95% CI)
Feltgen, N. et al. [27]	Case-by-case analysis (descriptive case-only study) population-based Case–control study cohort study	421 retinal vascular disease patients	28 days	an adjusted conditional logistic regression analysis was conducted Gutenberg Health Study	In this analysis, we compared the odds of being vaccinated in the last four weeks among patients with RVOD (cases) to controls from the general population recruited by the Gutenberg Health Study (GHS) (age ±5 years and sex-matched).	adjusted for obesity (BMI ≥ 30), diabetes, arterial hypertension, smoking, and use of anticoagulation.	Age: 67.6 ± 14.6 years; Sex: 51.8% female; 49.0% Eye (OS). A substantial cardiovascular risk profile in the cohort.	/	ChAdOx1 (AstraZeneca), BNT162b2 (Pfizer), mRNA-1273 (Moderna), Ad26.COV2 S (Janssen)	/	/	OR = 0.93; 95% CI: 0.60–1.45, *p* = 0.75

CI calculated using Clopper–Pearson exact method for binomial proportions.

Two European studies from Germany and Italy conducted multicenter self-controlled retinal vascular occlusion case series that may be associated with the COVID-19 vaccine and integrated baseline data from local populations. Feltgen et al. found no increased RVO risk 4 weeks post-vaccination (OR 0.93; 95% CI 0.60–1.45, *p* = 0.75) [27]. Pellegrini et al. reported no significant risk of RVO after the first dose (1–28 days IRR: 0.94, 95% CI 0.55–1.58) or second dose of the four vaccines (1–28 days IRR: 1.16, 95% CI 0.70–1.90) [26]. Both analyzed adenovirus vector vaccines (ChAdOx1 nCoV-19, Ad26.COV2.S) and mRNA vaccines (BNT162b2, mRNA-1273), finding no vaccine type-specific associations. However, their limited sample sizes (hundreds of cases) reduced statistical power.

Given RAO’s lower prevalence, few studies focus solely on RAO, often combining it with RVO analyses. For instance, Feltgen et al. included 118 RAO cases [27] and Hwang et al. included 6590 [22], both concluding no association with vaccination. However, Park et al. reported a higher cumulative RAO incidence with ChAdOx1 (0.0018%) [23]. Notably, the HR for RAO in women vaccinated with mRNA-1273 was 4.65 (95% CI 1.27–17.03; *p* = 0.02), warranting further investigation.

It is noteworthy that two studies using the TriNetX global network database reached opposing conclusions regarding mRNA vaccines. Li et al. [24], after excluding cases with a confirmed COVID-19 diagnosis or the use of specific medications (antiplatelet, anticoagulant, diuretic, contraceptive, antihemorrhagics), analyzed 4,619,499 individuals, matching vaccinated (BNT162b2 or mRNA-1273) and unvaccinated cohorts 1:1 by age and RVO risk factors. Using Cox multivariate and Kaplan–Meier analyses, they reported a 2.19-fold higher risk of retinal vascular occlusion in the vaccinated cohort over 2 years (95% Cl 2.00–2.39), with significantly higher cumulative incidence at 12 weeks and 2 years (log rank *p* < 0.001). Meanwhile, Dorney et al. [25] examined over 3 million individuals vaccinated with mRNA COVID-19 vaccines (Group 1), propensity score-matching them with influenza (Group 2) and Tdap (Group 3) vaccine recipients from 2018 to 2019 to assess the relative risk (RR) of new RVO diagnoses within 21 days. They drew the conclusion that there was no significant difference in RR post-COVID-19 vaccination compared to influenza (RR 0.74, 95% CI 0.54–1.01) or Tdap (RR 0.78, 95% CI 0.44–1.38) vaccination.

These conflicting findings have sparked debate. Both studies used the TriNetX database for 2020–2022 RVO cases and applied propensity score matching via the nearest neighbor greedy algorithm. However, control group selection, exclusion criteria and time windows selection differed significantly. Li et al. chose unvaccinated individuals from the same period, while Dorney et al. selected influenza and Tdap recipients from 2018 to 2019. COVID-19 infection’s impact on coagulation is a known risk factor for RVO. Dorney et al. did not exclude infected cases, despite the U.S. Centers for Disease Control and Prevention estimating a 43.9% infection rate. Li et al., relying solely on positive test records in TriNetX, may not have fully excluded asymptomatic or untested cases. Utilizing linked health claims and vaccination registries, Hashimoto et al. [28] assessed post-vaccination ocular events in Japan while excluding COVID-19-infected individuals. Their matched cohort analysis identified elevated RVO and composite outcome risks post-dose two, whereas self-controlled case series (SCCS) revealed no significant association for any ocular event. Additionally, Dorney et al. included patients on anticoagulants, antiplatelets, contraceptives, diuretics, and other medications without matching for these factors, potentially confounding coagulation status. Their observation windows also differed; Dorney used 21 days, while Li examined 12 weeks and 2 years. A shorter window aligns with case reports averaging a 2-week diagnostic interval. Longer periods during a pandemic may introduce confounding from repeated COVID-19 exposures, vaccination updates, or interim health changes. Extended windows may also detect delayed autoimmune responses, which clinical evidence suggests manifest after six weeks or more.

These studies face limitations due to RVO and RAO’s multifactorial nature. Firstly, accounting for all contributing factors is challenging, with key variables like ethnicity and ocular comorbidities often omitted. Secondly, the rarity of these events limits study power, particularly in smaller European cohorts. Thirdly, despite propensity score matching, residual imbalances in cardiovascular risk factors (e.g., age, hypertension, dyslipidemia) may bias results. Designing rigorous cohort studies during a pandemic with rapid viral transmission and complex clinical scenarios remains difficult.

Evidence shows temporal heterogeneity in RVO risk post-vaccination. Studies with ≤4-week windows typically report no association. A 60-day analysis found higher RVO incidence in vaccinated vs. unvaccinated cohorts (median diagnosis latency: 38 days). Li et al.’s extended observation (12 weeks to 2 years) uniquely identified vaccination as an RVO risk factor. This raises the question, do vaccine side effects emerge over time, increasing RVO incidence, as Li et al. suggest? Further research is needed.

Vaccine platforms exhibit safety profile heterogeneity for RVO risk. Kim et al. [21] reported a 4–6-fold higher RVO risk with ChAdOx1 compared to mRNA vaccines in a case–control analysis. Park et al. [23] reported significant sex-based differential risks; mRNA-1273-vaccinated females had a markedly elevated RAO hazard (aHR 4.65, 1.27–17.03; *p* = 0.02), whereas BNT162b2-vaccinated males showed reduced RVO incidence (aHR 0.66, 0.49–0.88; *p* = 0.004). However, most population-based studies found no significant platform-specific differences.

Emerging data suggest that most RVO cases occur after the first dose. Dorney et al. reported an RR of 2.25 (95% CI 1.33–3.81) for RVO after the first vs. second dose [25]. Park et al. found that 77% (633/822) of cases emerged post-first dose [23]. Günalp Uzun et al. noted a low VITT recurrence risk (3%) with mRNA boosters after ChAdOx1 priming [29]. Pellegrini et al. [26] demonstrated distinct dose–response patterns; adenovirus-vectored vaccines showed higher first-dose IRR at 4 weeks (1.61, 95% CI 0.57–4.55) versus second dose, while mRNA vaccines exhibited elevated second-dose IRR (1.12, 95% CI 0.52–2.38) compared to initial administration. This may reflect differences in the pathogenic principles of the two types of vaccines.

For patients with potential vaccine-related RVO, decisions on revaccination should consider systemic inflammatory markers (ESR/CRP), coagulation markers (D-dimer, anti-PF4 antibodies), fundus perfusion, and visual recovery.

## 3. Pathological Processes

**Figure 2:**
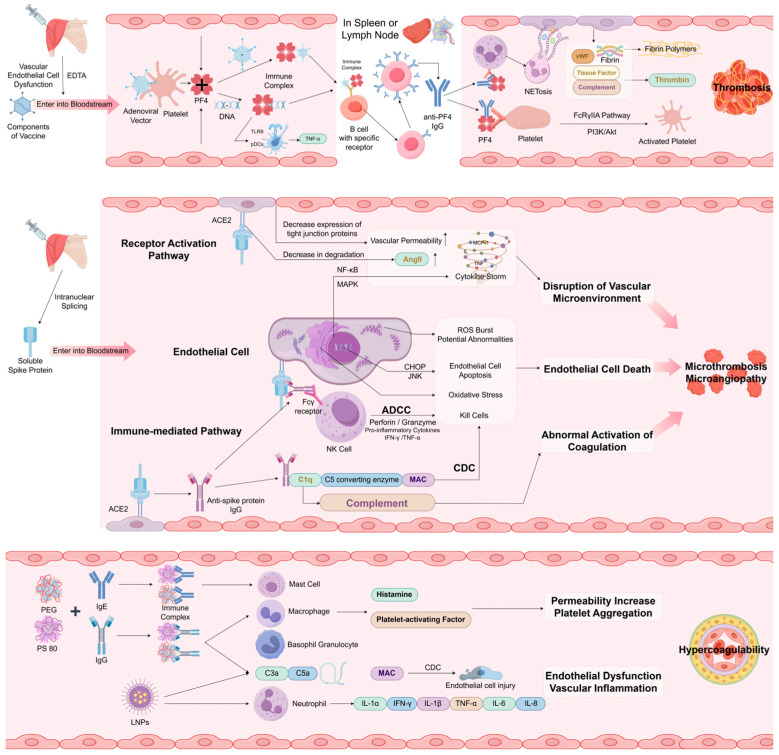
The schematic diagram illustrates currently proposed hypotheses of pathophysiological mechanisms associated with thromboembolic events or vasculopathic responses following administration of adenoviral vector-based and mRNA COVID-19 vaccines. This image was drawn using Figdraw.

The mechanisms linking COVID-19 vaccines to retinal vascular occlusion remain incompletely understood. For delayed central retinal vascular occlusion, vaccine-induced immune thrombotic thrombocytopenia (VITT) may be implicated. Peripheral occlusion within two weeks of vaccination may stem from immune responses to spike proteins or adjuvants, cytokine storms from allergic reactions, or vasculitis, mimicking the Virchow triad (vascular wall damage, blood stasis, hypercoagulability). We propose the following mechanisms based on observed blood abnormalities and pathophysiological evidence (Figure 2).

### 3.1. PF4 and VITT

Platelet factor 4 (PF4), or chemokine (C—X—C motif) ligand 4 (CXCL4), is abundant in platelets (20 μg/10^9^ platelets) with plasma levels of 2–10 ng/mL [30,31]. At physiological pH, PF4 exists as a highly cationic tetramer with extensive positive charge [32]. Pre-pandemic research on heparin-induced thrombocytopenia (HIT) showed that heparin, a polyanion, binds PF4, triggering anti-PF4-heparin antibodies that activate platelets, monocytes, neutrophils, and endothelial cells, leading to thrombocytopenia [33]. During mass vaccination, adenoviral vector vaccines, particularly ChAdOx1, have been linked to VITT [34]. Laboratory evidence suggests that adenoviral DNA may act as a polyanion, initiating VITT in susceptible individuals.

The VITT process may unfold as follows. Within a week of vaccination, endothelial injury from needle puncture or disruption of tight junctions by vaccine components (e.g., EDTA) may allow vaccine entry into the bloodstream [35]. Platelets may be activated by injection-related vascular injury, adenoviral binding to platelet surfaces, or immune complexes of host cell proteins and IgG antibodies. Adenoviral particles bind PF4 from activated platelets or the endothelial matrix [36,37]. These PF4-vaccine complexes are transported to the spleen, where B cells with PF4-specific Ig receptors proliferate, releasing high-titer anti-PF4 IgG within a week. FcRγIIA-mediated signaling of PF4-anti-PF4 IgG complexes induces pro-coagulant thrombocytosis, platelet/neutrophil aggregation, and NETosis. NETosis-released DNA amplifies immune damage, activating complement deposition in the endothelium. Activated endothelial cells express tissue factor and release von Willebrand factor (VWF), which binds activated platelets and clotting factors, further promoting neutrophil activation and thrombin generation, culminating in thrombosis [38].

An alternative hypothesis posits that circulating PF4 interacts with negatively charged extracellular DNA at the vaccination site, forming immune complexes that hyperactivate plasmacytoid dendritic cells (pDCs) via TLR9, boosting interferon-α production. RNA elicits a weaker effect. Memory B cells in regional lymph nodes then increase autoantibody production against PF4, perpetuating a cycle of FcRγII-mediated platelet activation and severe thrombosis.

The primary initiator of these mechanisms remains unclear, but adenoviral vaccines, containing both DNA and protein components, are the most implicated in VITT. ChAdOx1’s high electronegative surface potential enhances its interaction with oppositely charged proteins like PF4 [36]. Nucleic acids bind PF4 when plasma concentrations exceed 200 ng/mL, though vaccine-derived nucleic acids typically remain below this threshold [39]. Electron and super-resolution microscopy indicate that PF4-anti-PF4 VITT antibody complexes primarily bind nonstructural vaccine components (e.g., host cell proteins, unassembled hexons), amplified by DNA [40].

Thrombosis and thrombocytopenia in VITT are mediated by distinct mechanisms. Anti-PF4-polyanion antibodies drive platelet and neutrophil activation, with thrombocytopenia potentially resulting from direct platelet binding [38]. VWF knockout mice showed no significant thrombocytopenia post-adenovirus, suggesting VWF and P-selectin mediate platelet clearance [37]. NETosis, driven by neutrophil activation, is a key contributor to VITT thrombosis, with inhibition of NETosis (via GSK484 or PAD4 knockdown) preventing thrombosis but not thrombocytopenia. In platelet absence, PF4 immune complexes can directly activate neutrophils. Despite its prominence, VITT remains rare, with anti-PF4-polyanion antibodies typically low in optical density and not inducing abnormal platelet activation in most vaccinated individuals [41].

### 3.2. Spike Protein

SARS-CoV-2, an RNA virus, completes its lifecycle in the cytoplasm, lacking evolutionary pressure for splice donor (SD) and acceptor (SA) sites. Vaccines based on mRNA technology, whose functional activity is limited to the cytoplasm, differ fundamentally from adenoviral vector vaccines, where transcription occurs in the nucleus and undergoes RNA splicing [42]. Experimental data show that adenoviral vector vaccines can produce soluble spike protein variants due to intranuclear splicing, allowing these proteins to enter the bloodstream [42]. However, free spike antigen was detected in the blood of adolescents and young adults who developed post-mRNA vaccine myocarditis [43]. Soluble spike proteins may trigger signaling pathway abnormalities through direct binding to receptors or through immune-mediated cell injury, ultimately leading to microangiitis and microthrombosis.

We hypothesize that soluble spike proteins bind angiotensin-converting enzyme 2 (ACE2) via their receptor-binding domains (RBDs), with anti-spike antibodies recognizing these variants on endothelial cells [44,45]. This may trigger local inflammation through (1) cytokine/chemokine induction (e.g., IL-6, IL-1β, TNFα, CXCL1, CXCL2, CCL2); (2) antibody-dependent cell-mediated cytotoxicity (ADCC), where NK cells target endothelial cells via Fcγ receptors [46,47]; and (3) complement-dependent cytotoxicity (CDC), where complement activation promotes membrane attack complex MAC formation, platelet activation, and thrombin release [48].

Spike proteins impair endothelial function by reducing nitric oxide synthase activity and mitochondrial function, increasing redox stress, and destabilizing ACE2, potentially exacerbating endothelial dysfunction via the renin-angiotensin system [49]. Although genetically modified to enhance immunity and prevent ACE2 binding, soluble spike proteins may still induce inflammatory responses in endothelial cells, mimicking the thromboembolic effects of wild-type SARS-CoV-2 or pseudoviruses [50].

These variants may explain rare adverse events post-Vaxzevria (ChAdOx1) vaccination. Unlike Ad5.S and ChAdOx1.S, Ad26.COV2. S lacks key splice sites (SD506, SD3614), with no significant spliced RNA detected, suggesting lower risk [42]. However, evidence indicates platelets and megakaryocytes lack ACE2, implying alternative entry mechanisms (e.g., TLR7) for SARS-CoV-2 or spike proteins, requiring further investigation [50], such as through transferrin receptor [51].

### 3.3. Allergic Reactions or Vasculitis Due to Adjuvants

Adjuvants in vaccines enhance stability and efficacy but may contribute to rare thrombotic events. Though RVO reports linked to adjuvants are scarce, hypotheses suggest immune/allergic reactions or small-vessel vasculitis. Polyethylene glycol (PEG) in mRNA vaccines (BNT162b2, mRNA-1273) may bind anti-PEG antibodies (prevalence 25% vs. 0.2% historically [52,53]), activating complement (C3a, C5a) and inflammatory cells (macrophages, basophils, mast cells), releasing mediators like histamine and platelet-activating factor [54,55]. The ChAdOx1 nCov-19 and Ad26. COV. 2 vaccines contain polysorbate 80, a synthetic nonionic surfactant and emulsifier that is widely utilized to fabricate nanoparticle drug delivery platforms. Structurally, polysorbate 80 resembles PEG, featuring repetitive ethylene oxide sidechains that may elicit analogous allergic reactions [56]. In preclinical studies, polysorbate 80 enhances membrane permeability and disrupts the blood–brain and intestinal barriers [57]. However, no empirical evidence currently suggests analogous effects on the blood-retinal barrier, which is structurally similar. EDTA in ChAdOx1 may activate platelets and cause capillary infiltration, though its low dose (500 µL intramuscularly) makes systemic effects unlikely [58].

Lipid nanoparticles (LNPs) in mRNA vaccines, comprising phospholipids, cholesterol, PEG-lipids, and ionizable lipids, exhibit adjuvant activity, protecting mRNA and aiding delivery [59]. In mice, LNPs trigger neutrophil infiltration, inflammatory pathways, and cytokine/chemokine production (e.g., IL-1β, IL-6) [60]. Studies show that Pfizer’s Comirnaty and Moderna’s Spikevax increase C5a, sC5b-9, and Bb in serum, with Comirnaty inducing pro-inflammatory cytokines (IL-1α < IFN-γ < IL-1β < TNF-α < IL-6 < IL-8) in PBMC cultures [61]. These responses may cause transient endothelial dysfunction and inflammation, particularly after the second dose [62], predisposing vessels to thrombosis [63,64,65,66].

### 3.4. Molecular Mimicry

Molecular mimicry, where vaccine components resemble human proteins, may trigger autoimmunity in susceptible individuals [67]. Computational analysis identified 5693 epitopes in BNT-162b2 corresponding to 21 viral and human proteins, some linked to immune diseases via HLA alleles [68]. However, no direct evidence connects molecular mimicry to retinal vascular occlusion.

### 3.5. Why Thrombosis Occurs in Retinal Vessels

**Figure 3:**
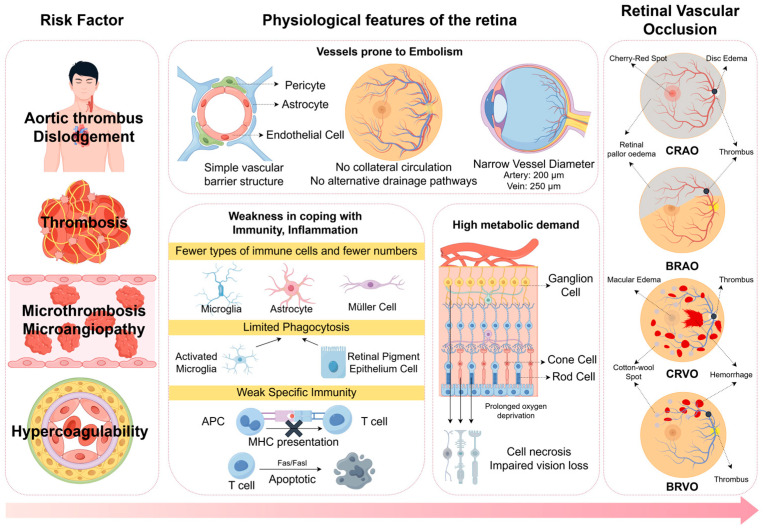
This figure delineates the pathophysiological mechanisms underlying hemodynamic disturbances and neurovascular architecture deconstruction in the delicate retinal tissue, mediated by systemic vascular risk factors, culminating in vision loss. This image was drawn using Figdraw.

Retinal vessels may be particularly susceptible to vaccine-related thrombosis due to their unique anatomy and physiology. The retina’s high metabolic demand and limited collateral circulation amplify the impact of vascular occlusion. Endothelial cells in retinal capillaries express ACE2, potentially interacting with soluble spike proteins or immune complexes [49]. The Virchow triad—exacerbated by vaccine-induced endothelial damage, stasis from inflammation, and hypercoagulability from VITT or adjuvants—may preferentially manifest in these small-caliber vessels. Additionally, the retina’s immune-privileged status may delay clearance of inflammatory mediators, prolonging thrombotic risk [69]. Further studies on retinal-specific factors are needed (Figure 3).

Due to data volume limitations, big data studies do not visually analyze retinal conditions through fundus imaging; they often use codes in the system to confirm diagnoses. In this regard, there are small cohort studies that have analyzed retinal perfusion after vaccination [70,71,72]. Using the optical coherence tomography angiography (OCTA) in individuals vaccinated with the Pfizer-BioNTech vaccine, Birumut Gedik et al. [70] found a significant decrease in vessel density and a significant increase in retinal thickness. However, in patients vaccinated with inactivated virus vaccine, no such changes in retinal structure were observed.

## 4. Discussion

In the post-pandemic era, researchers are exploring whether reinfection or multiple infections post-vaccination elevate complication risks. Current focus examines if residual vaccine components (nucleic acids, adenoviral vectors, spike proteins) amplify immune responses during subsequent SARS-CoV-2 infections. Notably, VITT-related anti-PF4 antibodies do not cross-react with viral spike proteins [73], and COVID-19-associated thrombosis operates distinctly from VITT pathways [67]. Park et al.’s subgroup analyses [23] showed higher RAO and RVO risks in individuals vaccinated before COVID-19 diagnosis compared to those vaccinated post-diagnosis or unvaccinated, though risks remained lower than in unvaccinated, uninfected individuals. This suggests no excessive concern for vaccine-COVID-19 interactions is warranted. In contrast, other analysis [5] indicates a significant increase in the incidence of retinal vein occlusions (RVOs) within 6 months post-COVID-19 diagnosis (adjusted IRR, 1.54; 95% CI, 1.05–2.26; *p* = 0.03). Although retinal artery occlusions (RAOs) showed a nominally elevated incidence (adjusted IRR, 1.35; 95% CI, 0.64–2.85; *p* = 0.44), this did not reach statistical significance. Critically, COVID-19 vaccination demonstrates well-established protective effects against severe respiratory complications (e.g., acute respiratory distress syndrome) and cardiovascular sequelae following SARS-CoV-2 infection. While the link between vaccination and fundus blood flow issues remains inconclusive, COVID-19 itself poses a far greater risk than vaccines.

The clear conclusion we can draw is that cases of retinal vascular occlusion following vaccination are rare. In our review, we found that most of the cases had common risk factors for retinal vascular occlusion such as hypertension and hyperlipidemia. Individuals at particularly high risk should be informed of this risk before vaccination. At the same time, these individuals should be protected by routine visual acuity examinations after vaccination.

Case reports noting short intervals between COVID-19 vaccination and RVO onset likely reflect reporting bias, with clinicians attributing proximate events to vaccination absent robust pathological evidence. Molecular and animal studies suggest adenoviral vector-induced VITT manifests > 14 days post-vaccination. Many studies fail to distinguish vaccine types, despite their differing compositions and mechanisms. Future case reports should document inflammatory markers and anti-PF4 antibodies, analyzing onset intervals and the presence or absence of COVID-19 infection meticulously.

Based on compositional and pathogenic mechanisms, the immunogenicity of mRNA vaccine components—specifically spike proteins and LNP carriers—may induce vascular tissue damage and trigger thrombosis. However, as noted previously, ChAdOx1 contains adenoviral vectors with higher pathogenic potential and more frequent RNA intranuclear splicing, potentially leading to greater clinical incidence. At the level of patient-derived antibodies, the striking similarity in autoantibody fingerprints between these two conditions strongly indicates that VITT and adenovirus infection-associated anti-PF4 disorders represent a distinct category of adverse immune responses targeting adenoviral structures [74]. While minimal differences exist for retinal vascular occlusion, ChAdOx1 demonstrates comparatively higher risks in other immune-mediated and thrombotic diseases versus mRNA vaccines [75].

Four years after the COVID-19 vaccination campaigns, sporadic reports describe patients experiencing prolonged symptoms lasting 12 months or longer following acute post-vaccination reactions [76,77,78]. These observations suggest potential long-term vaccine-associated effects, though establishing causality faces significant challenges. This diagnostic uncertainty underscores the need for internationally accepted criteria to define “long post-COVID-19 vaccination syndrome”. Standardized enrollment protocols would enable systematic global data collection and analysis.

In addition to this, we need more high-quality studies to analyze the incidence of long-term disease after COVID-19 vaccination, such as the article by Li et al. [24]. Such studies will clarify whether vaccine-related complications have the potential for delayed onset. Concurrently, we must assess if biological mechanisms exist for delayed-onset vaccine-related manifestations. A novel disease entity termed VITT-Like Monoclonal Gammopathy of Thrombotic Significance (VITT-like MGTS) has been proposed. This condition is characterized by M-protein production and the presence of VITT-like autoantibodies in serum/plasma. Crucially, the M-protein itself exhibits platelet-activating properties analogous to those in classical VITT [79]. Patients present with chronic, progressive, or intermittent thrombocytopenia and develop a chronic prothrombotic hypercoagulable state, leading to recurrent systemic thrombosis. The disease course may persist for years. Whether retinal vascular thrombosis represents a clinical manifestation of VITT-like MGTS remains unestablished and warrants further investigation [80].

Investigating the association between retinal vascular occlusion (RVO) and COVID-19 vaccines extends beyond observational studies, with prospective interventional trials representing a critical methodological avenue—though none have been published to date. Given RVO’s multifactorial pathophysiology and heterogeneous progression, adaptive trial designs offer distinct advantages. By actively assigning interventions (e.g., vaccine platform, dosing intervals, sample size re-estimation) while controlling variables, such designs directly quantify causal effects and minimize residual confounding.

Within retrospective frameworks, propensity score weighting—particularly inverse probability treatment weighting (IPTW) coupled with machine learning algorithms—has emerged as a key technical approach for handling high-dimensional confounders. IPTW assigns each subject a weight to construct a pseudo-randomized population where measured covariates achieve inter-group balance, simulating randomized trial conditions. Machine learning techniques (e.g., LASSO regression, random forests, gradient boosting machines) enable robust propensity score estimation through effective processing of numerous covariates—including nonlinear relationships and interactions—while performing variable selection to reduce noise.

The self-controlled case series (SCCS) method may provide longitudinal advantages for pathophysiological timeline investigation. However, this design carries limitations; it may fail to address within-individual temporal confounding from time-varying factors (e.g., healthcare utilization, medication changes, or disease progression), potentially leading to vaccine-attribution bias. Consequently, SCCS application requires presuming transient, acute vaccine effects on RVO. Prolonged risk windows amplify time-varying confounding, making precise definition of risk/control window durations challenging yet essential.

In the context of the post-epidemic era, this paper systematically reviews the association between COVID-19 vaccination and the risk of retinal vascular occlusion (RVO and RAO) with the aim of integrating the existing evidence, dissecting the underlying thrombotic mechanisms. Building upon prior studies, our study reveals the mechanisms that may cause vascular occlusion by direct comparative analysis of the biological components of adenoviral vectors and mRNA vaccine platforms. These findings directly inform clinical protocols for monitoring high-risk subgroups, such as individuals with anti-PF4 antibody profiles. These findings do not go to blame the vaccines that have made a significant contribution to controlling the COVID-19 pandemic, but rather to inform approaches for next-generation vaccine development [81]—including the rational design of low-immunogenicity delivery vectors and adjuvant systems [82,83]—to optimize the safety and efficacy of vaccination programs in preparation for possible future pandemics.

## 5. Conclusions

Similarly to other systemic vasculopathies, current evidence does not establish a definitive association between COVID-19 vaccines and retinal vascular occlusions in the short term. Due to the lack of research evidence, we cannot jump to conclusions about the effects of vaccines within a long window of time. For patients with predisposing factors such as hyperlipidemia or hypertension, comprehensive hemodynamic evaluations should precede vaccination to mitigate potential risks associated with Virchow’s triad. Post-pandemic surveillance of visual acuity changes remains imperative for early detection of vaccine-related or COVID-19-related ophthalmic sequelae.

Big data analytics face inherent challenges in investigating retinal vascular occlusions due to their low incidence rates, multifactorial etiology, and confounding from widespread COVID-19 infections. Given the near-universal exposure to COVID-19 vaccines and the challenges of studying rare outcomes like retinal vascular occlusion (RVO) with multiple confounders, no single methodological approach is flawless. We must strategically combine complementary study designs to mitigate their respective limitations. Longitudinal investigations of pathophysiological timelines, either after COVID-19 infection or after vaccination, will optimize critical observation window selection for causal inference.

## Abbreviations

The following abbreviations are used in this manuscript:

RVO	Retinal vascular occlusion
RAO	Retinal artery occlusion
OR	Odds ratios
HR	Hazard ratio
RR	Relative risks
VITT	Vaccine-induced immune thrombotic thrombocytopenia
PF4	Platelet Factor 4
ACE2	Angiotensin-converting enzyme 2
SARS-CoV-2	Syndrome-coronavirus-2
CI	Confidence interval
EDTA	Ethylenediaminetetraacetic acid
VWF	Von Willebrand factor
pDCs	Plasmacytoid dendritic cells
SD	Splice donor
SA	Splice acceptor
RBDs	Receptor-binding domains
ADCC	Antibody-dependent cell-mediated cytotoxicity
CDC	Complement-dependent cytotoxicity
MAC	Membrane attack complex
OCTA	Optical coherence tomography angiography
